# Socio-demographic factors associated with overweight and obesity among primary school children in semi-urban areas of mid-western Nigeria

**DOI:** 10.1371/journal.pone.0214570

**Published:** 2019-04-03

**Authors:** Adewale Elijah Adetunji, Kayode Adesoye Adeniran, Sylvia Chinwendu Olomu, Angela Ifeoma Odike, Rosemary Omonigho Ewah-Odiase, Irekpono Ukhueleigbe Omoike, George Obozokhale Akpede

**Affiliations:** 1 Department of Paediatrics, Irrua Specialist Teaching Hospital, Irrua, Edo State, Nigeria; 2 Department of Paediatrics, Federal Medical Centre, Asaba, Delta State, Nigeria; 3 Department of Paediatrics, Faculty of Clinical Sciences, College of Medicine, Ambrose Alli University, Ekpoma, Edo Sate, Nigeria; Loyola University Chicago, UNITED STATES

## Abstract

**Background:**

Many developing countries are in a state of nutritional transition from prevalent under-nutrition to the emergent problem of over-nutrition (overweight and obesity), which is associated with increased morbidity and mortality, and whose complications can persist into adulthood with long-term consequences. However, data are limited on the risk factors for overweight and obesity (O&O) among primary school children, particularly those in rural and semi-urban areas in these countries.

**Aim and objectives:**

To determine the socio-demographic factors associated with overweight and obesity among primary school children in semi-urban areas.

**Subjects and methods:**

1187 school pupils aged 6–11 years recruited from semi-urban areas using multistage sampling were interviewed for risk factors of overweight and obesity using a structured questionnaire. Nutritional status was assessed using body mass index and this was classified using a standard method. The proportions were compared using Pearson’s chi-squared. Multivariate logistic regression analysis was also carried out with overweight and obesity as the dependent variable and socio demographic factors as independent variables. The level of statistical significance was set at *p* <0.05 in all the statistical analyses.

**Results:**

Fifty-eight pupils (4.9%) had overweight and obesity while 1129 (95.1%) were either of normal nutritional status (1088, 91.6%) or were thin/severely thin (41, 3.5%). Among those with overweight and obesity, 41 (3.5%) were overweight and 17 (1.4%) obese. A higher prevalence of overweight and obesity was significantly associated (in unadjusted analysis) with female gender [unadjusted Odds Ratio, OR (95% CI) = 2.42 (1.37, 4.28)], attendance at private schools [OR (95% CI) = 3.34 (1.86, 6.00)], higher socio-economic status families [OR (95% CI) = 2.32 (1.65, 5.80)] and presence of a television in the pupil’s bedrooms [OR (95% CI) = 2.22 (1.02, 4.82)] on bivariate analyses. However, only gender, school type and family socioeconomic status were independently associated with overweight and obesity on multivariate logistic regression analysis.

**Conclusion:**

We conclude that overweight and obesity among primary school pupils in semi-urban areas is associated with female gender, attendance at private schools and higher socioeconomic status families. Preventive programmes should accordingly be more directed at children from affluent families; particularly those who are females and those attending private schools.

## Introduction

Childhood overweight and obesity (O&O), an emerging public health problem in developing countries, has been linked with the increasing westernization of societies and associated changes in lifestyle [1]. Many developing nations are in a state of nutritional transition [2,3], in which there is a persistently high level of under-nutrition in the face of emerging O&O [2,3]. In Nigeria, and other African countries, the prevalence of childhood O&O has ranged from 0% to 26.7% in different age groups, depending on the methods of assessment used [4–13]. Examples of different methodologies are measurement of body mass index [4,6,7] versus bio-electrical impedance [5] versus waist circumference [8], and differences in definition viz World Health Organization 2007 [4,6] versus International Obesity Task Force [7] versus National Centre for Health Statistics [4].

The complications of O&O could occur during childhood and adolescence and persist into adulthood, with increased risk of morbidity and mortality later in life [14, 15]. These complications include the development of elevated blood pressure with its attendant risks of long-term cardiovascular morbidity and early death [16, 17]. On account of these complications, the prevention and treatment of O&O in childhood have emerged as an important focus of paediatric research and clinical care [15].

Environmental and genetic factors have been implicated in the prevalence of O&O [15,18,19], although some inconsistencies in the effects have been reported [20]. Among children especially in developing countries, being born into a family with a high income, higher levels of maternal education, low levels of physical activity, female gender and race are major risk factors. [21,22]. However, prenatal factors such as maternal gestational diabetes and fetal nutritional status are also important [15]. The pattern of physical activity, television viewing and family socioeconomic status could be interrelated but few studies in Nigeria have looked at the impact of presence or absence of television in the child’s sleeping room and number of hours spent per day on the screen on the prevalence of O&O. Furthermore, the reported effects of some risk factors like socioeconomic status of the family and race have been inconsistent [22–24].

The early identification of modifiable risk factors in childhood could provide an avenue for intervention in curbing the increasing prevalence of O&O [25]. O&O are more amenable to treatment in early life and early intervention could help to reduce both the short-term and long-term consequences of O&O, as well as break the tracking into adolescence and adulthood [26]. The aim of this study was to explore the risk factors associated with O&O among primary school age children in Esan West Local Government Area (EWLGA), Edo State, Nigeria, EWLG is a semi-urban area with a total of 16 communities organized under 10 political wards with the administrative headquarters at Ekpoma, which is located about 80km from Benin City, the Capital of Edo State [27]. The inhabitants of the LGA are mostly farmers, but there are also traders, civil servants and staff of tertiary health and educational institutions [27, 28]. A semi-urban area is an area that is undergoing transition from its rural to urban lifestyles without leaving the original environment [29]. Most of the inhabitants are individuals who have low socio-economic status being mainly agricultural workers, artisans and traders, and are resident in areas that often lack basic infrastructures such as pipe borne water and good roads. However, compared to life in typical rural communities, the inhabitants have adapted to Western-oriented lifestyles [30], although with a less intense exposure to the electronic and print media and sedentary lifestyles compared with urban areas [27, 28].

## Methodology

The study was a cross-sectional survey of primary school pupils carried out from June to September 2013 in Esan West Local Government Area (EWLGA), Edo State, Nigeria. EWLGA is in socioeconomic transition, with large parts changing from rural to semi-urban [27, 28].

The pupils were recruited using multistage random sampling. Three political wards were first randomly selected from the 10 wards in the LGA. Eleven registered primary schools, representing 6 (13.3%) of the 45 public schools and 5 (20%) of the 25 private schools in the LGA were then randomly selected from the three wards. One arm per school class was thereafter randomly selected by balloting from each school, and all the pupils in the selected arm who met the inclusion criteria were included in the study as described in earlier studies [19, 31].

One thousand, three hundred and ten pupils aged 6–11 years and in primary classes 1–6 were recruited initially but only 1187 (90.6%) were included in the analysis. The remaining 123 (9.4%) were excluded because their parents/guardians did not give consent (n = 93) or because of incomplete data (n = 30).

The Research and Ethics Committee of Irrua Specialist Teaching Hospital (ISTH) approved the study protocol. Approval was also obtained for the study from EWLGA Education Authority while written informed consent was obtained from the parents/guardians of the children, and permission was taken from the Headmasters and Headmistresses of participating schools. In addition, the pupils also gave verbal assent after they were duly informed and educated about the importance of the study and the procedures involved.

The study instrument consisted of a questionnaire, which was filled out by the parents/guardians. The information obtained included socioeconomic status of the pupil’s parents and the pupil’s age as at last birthday among others. The questionnaire and consent forms were sent to the parents/guardians of the pupils through the pupils, under the supervision of the class teachers. Those parents/guardians who gave consent filled the questionnaire, signed the consent forms and returned them within three days to the class teachers. Those who declined consent also returned both the unfilled questionnaire and consent forms indicating their refusal to allow participation of their children. All the pupils with an abnormal nutritional status (overweight, obesity, thinness and severe thinness) were referred to ISTH for further evaluation.

Two Research Assistants were recruited for the study from among House Officers in the Department of Paediatrics, ISTH and trained for one day on the measurement of weight and height, and the procedure for obtaining verbal assent from the pupils. They were also taught how to protect the privacy of the pupils and to identify those in need of urgent medical attention. One Research Assistant took the height while the other took the weight throughout the study. This was in order to avoid inter-observer variations in the measurements.

Nutritional status was assessed using the Body Mass Index (BMI), which was calculated using the formula, weight (in kg)/(height)^2^ in m^2^. Weight was measured using Seca digital electronic scale, with the pupils barefooted, clothed in light sportswear but without a belt and wristwatch, after urination [32]. With the scale placed on a level solid floor, the pupils stood on it with feet placed together and the weight was read to the nearest 0.1 kg after the displayed reading had become stable. The scale was calibrated using 6kg dumb-bells. Height was measured to the nearest 0.1 cm with a portable Prestige Stadiometer. The pupil stood erect with the bare feet kept together and the occiput, shoulders, buttocks and the heels placed against the perpendicular part of the stadiometer. During the procedure, the pupil looked straight ahead with the external auditory meatuses on the same horizontal plane as the lower borders of the eye sockets, the Frankfort horizontal plane [32]. Gentle pressure was then applied upwards to the bony prominences just behind the ears to stabilize the head while the headrest was then brought to rest on the head of the pupil and the pointer read off. The measurements of weight and height were carried out in the school clinic/sick bay or school library where there was no sick bay, in the presence of the class teacher. This was to ensure compliance with study protocol and conditions of approval by EWLG Educational Authority, especially that no blood samples are drawn from the children and that they are not given any thing to eat. Nutritional status was classified based on BMI into obesity (BMI >+2 SD), overweight (BMI >+1SD to ≤+2SD), normal (BMI = ˗2SD to +1SD), thinness (BMI <˗2SD to ≥˗3SD) and severe thinness (BMI <˗3SD) using the 2007 WHO (World Health Organization) growth reference charts into [33].

The socio-economic class of the families was determined using Oyedeji’s method [34], in which socio-economic class is the average of the highest educational attainment and occupation of the mother and father. The educational status of each parent was given a score of 1 to 5 depending on the level attained (University graduate or equivalent = 1; School certificate holder or equivalent = 2; Grade II teacher or equivalent = 3; Primary school certificate holder = 4; and No formal education = 5). The occupation of each parent was also given a score of 1 to 5 depending on the specific job (Professionals, senior public servants, owners of large business concerns = 1; Non-academic professionals such as secondary school teachers and confidential secretaries = 2; Non-manual skilled workers including clerks, typists, and telephone operators = 3; Petty traders, labourers and messengers = 4; and Unemployed, full time house wives and students = 5). The mean of four scores, two (educational attainment and occupation) for the father and two (educational attainment and occupation) for the mother, to the nearest whole number was taken as the socio-economic score of the family. This gave score 1, 2, 3, 4,and 5 corresponding to socioeconomic classes I, II, III, IV and V respectively, which were then classified into higher (classes I-III) and lower (classes IV and V) classes as recommended [34]. Screen time was defined as the time spent watching television, video games or computer and was measured as the average number of hours per day spent on screen [15].

## Data analysis

Data were entered into Microsoft Office Excel 2007 and analysed using Open Epi Info Version 3.01, and SPSS version 20. For ease of analysis, variables were categorized into groups based on age, nutritional status, and socioeconomic status. Age was classified into early (6–8 years old) versus late (9–11 years old) school age. Nutritional status was classified into overnutrition (overweight and obesity) versus normal/under nutrition (thinness and severe thinness) and socioeconomic status into higher (socioeconomic classes I-III) and lower (socioeconomic classes IV and V) status as already described.

Proportions were compared between groups using chi-squared test. Bivariate regression analysis was used to assess the relationship between O&O and each of the risk factors separately while multivariate logistic regression analysis was done to further assess the relationship between socio-demographic factors (independent variables) as determinants of the prevalence of O&O (dependent variable) while adjusting for confounding variables. The independent variables in the regression analysis were dichotomized. Other than screen time, all the variables (age, school type, socioeconomic status, gender and television in bedroom) in the bivariate regression were included as independent variables in the multivariate regression analysis and were all adjusted for. Screen time was excluded since the presence of a television in the bedroom was in the model and screen time is correlated with presence of a television. There was no significant correlation among other independent variables. However, a sub-analysis on the effect of screen time on the prevalence of O&O in the group of children with a television in their bedrooms was done.

The results of statistical analyses are presented in as odds ratios (OR) and 95% confidence intervals (CI). The level of statistical significance was set at *p* <0.05 in all the statistical analyses, which corresponds with an OR with a 95% CI that does not include zero (0).

## Results

### Socio-demographic characteristics of the pupils

Table 1 shows the socio-demographic characteristics of the pupils. The number of early school age pupils was slightly lower than that of late school age pupils (ratio = 0.9: 1) while the number of males was slightly higher than that of females (ratio = 1.1: 1). There were more pupils from public than private schools (ratio = 1.2: 1) and from higher than lower socioeconomic status families (ratio = 1.2: 1). A majority (92.9%) of the pupils had no television in their bedrooms and only 28.3% had ≥3 hours of screen time per day.

**Table 1:** Socio-demographic characteristics of the pupils.

Feature	Status	No.	Percentage (%)
**Age**	Early school age	559	47.1
	Late school age	628	52.9
**Gender**	Male	607	51.1
	Female	580	48.9
**School type**	Public	648	54.6
	Private	539	45.4
**SES of families**	Lower	546	46.0
	Higher	641	54.0
**TV in pupil’s bedroom**	Yes	84	7.1
	No	1103	92.9
**Screen time**	≥ 3 hours	336	28.3
	< 3 hours	851	71.7

SES = socioeconomic status, TV = television

### Nutritional status of the pupils

Fifty-eight pupils (4.9%) had O&O while 41 (3.5%) had under-nutrition (being thin/severely thin) and 1088 (91.6%) were of normal nutritional status. Among those with O&O, 41 (3.5%) were overweight and 17 (1.4%) obese.

### Association between the selected socio-demographic factors and the prevalence of overweight and obesity

Table 2 shows the association between the prevalence of O&O and the selected socio-demographic factors of the pupils. There was a significantly higher prevalence of O&O among female pupils (*p* = 0.002), and pupils from the higher socioeconomic status families (*p* <0.001) and private schools (*p* <0.001). The prevalence of O&O was also significantly higher among pupils with a television in their bedroom (*p* = 0.041). However, there was no significant difference between the prevalence of O&O and school age (early versus late, *p* = 0.089) or screen time (≥ 3 hours versus < 3 hours, *p* = 0.636).

**Table 2:** Association between the selected socio-demographic factors and the prevalence of overweight and obesity.

Factor	Status	No.	O&O No. (%)	Crude OR (95% CI)	*P*
**School age**	Late	628	37 (5.9)	1.60 (0.93,2.77)	0.089
	Early	559	21 (3.8)	1	
**Gender**	Female	580	40 (6.9)	2.42 (1.37, 4.28)	0.002*
	Male	607	18 (3.0)	1	
**SES of families**	Higher	641	45 (7.0)	2.32 (1.65, 5.80)	<0.001*
	Lower	546	13 (2.4)	1	
**School type**	Private	539	42 (7.8)	3.34 (1.86, 6.00)	<0.001*
	Public	648	16 (2.5)	1	
**TV in pupil’s bedroom**	Yes	84	8 (9.5)	2.22 (1.02, 4.82)	0.041*
	No	1103	50 (4.5)	1	
**Screen time**	≥ 3 hours	336	18 (5.4)	1.15 (0.65, 2.03)	0.636
	<3 hours	851	40 (4.7)	1	

O&O = overweight and obesity, OR (95% CI) = Odd Ratio (95% Confidence Interval), SES = socioeconomic status, TV = television

* Statistically significant at *p*<0.05.

### Multivariate logistic regression analysis showing association between O&O and socio-demographic factors

Table 3 shows the results of the multivariate logistic regression analysis of the association between the prevalence of O&O, and socio-demographic factors. School type (*p* = 0.006), family socioeconomic status (*p* = 0.019) and gender (*p* = 0.004) had a significant association with the prevalence of O&O.

**Table 3:** Socio-demographic factors and the odds of overweight and obesity among the pupils.

Factor	Adjusted OR (95% CI)	*p*
**Age**	0.707 (0.411, 1.217)	0.211
**School type**	2.409 (1.291, 4.495)	0.006*
**Socioeconomic status**	2.228 (1.142, 4.346)	0.019*
**Gender**	0.431 (0.242, 0.767)	0.004*
**Television in bedroom**	1.742 (0.778, 3.900)	0.177

OR = Odd Ratio, 95% CI = (95% Confidence Interval), O&O = overweight and obesity. All the variables in the model were adjusted for. Omnibus tests of model coefficients indicated that the χ2 value of the logistic regression model was 37.089 with a *p* < 0.001.

* Statistically significant at *p* <0.05.

### Sub-analysis of the effect of screen time on the prevalence of O&O in pupils with a bedroom television set

Among pupils with TV in the sleeping rooms, the prevalence of O&O was higher among pupils with screen time of ≥ 3 hours per day (16.0%) compared with those who spent less than 3 hours per day. However, the difference was not statistically significant. This is shown in Table 4.

**Table 4:** Sub-analysis of the effect of screen time on the prevalence of O&O in pupils with television in the bedrooms.

TV in bedroom	Screen time in hours	No.	No. (%) with O&O	Crude OR (95% CI)	*p*
**Yes**	< 3	59	4 (6.8)	1	
	≥ 3	25	4 (16.0)	2.62 (0.61, 11.20)	0.378
**No**	< 3	792	36 (4.5)	1	
	≥ 3	311	14 (4.5)	0.99 (0.53, 1.86)	0.975

TV = television, O&O = overweight and obesity, OR = Odd Ratio, 95% CI = 95% Confidence Interval.

## Discussion

The prevalence of O&O was 4.9% (overweight 3.5% and obesity 1.4%) in this study. This falls within the broad range of 0 to 26.7% in previous reports from within and outside Nigeria [4–13]. The prevalence rates of O&O are comparable with the 2.1% - 3.7% in most reports from urban, semi urban and rural areas of Nigeria [6,7,35,36], higher than the low rates of 0.0% - 1.9% in some reports from Southwestern Nigeria [4,31], and lower than the rates of 4.9% - 26.7% in some others [5,10,11,37] from within and outside Nigeria. Differences in methodology, study location (rural, semi-urban or urban) and selection criteria, could account for the differences in prevalence rates between studies.

This study was conducted to identify the socio-demographic and behavioral factors associated with the risk of O&O. As in previous reports [5–7,11,36,38,39], gender, SES, school type and availability of a TV in the pupil’s bedroom were individually associated with overweight and obesity. However, only gender, school type and SES of the family remained independently and significantly associated with overweight and obesity on multivariate analysis.

The effect of gender is in agreement with previous studies [5–7,11,36,38,39] and may be due to restriction of the physical activity of female children by parents [40,41]. Moderate physical activity reduces the risk of overweight and obesity and the associated complications [42].

The higher prevalence of O&O among pupils from the higher socioeconomic classes and those attending private schools confirms the results of previous studies from developing countries [7,11,31,43,44–47]. The effect might be related to the fact that higher socio-economic class parents commonly send their children to privately-run educational institutions in most developing countries [45]. However, the higher prevalence of O&O among pupils from the higher socio-economic class family is at variance with the results from developed countries in which minorities and Blacks have a higher prevalence of O&O [48–50]. Differences in lifestyle between developed and developing countries could account for the observed differences in prevalence patterns [51]. Richer families in developing countries are thought to have better access to meat and other energy-dense fast foods, which are more expensive than other foods like vegetables, than poor families [51]. The emerging market for fast foods in Nigeria, with its increasing popularity among children, has been linked to the increased risk of obesity [52,53]. The increasing popularity of fast food is due to its easy accessibility, sweet taste, parent’s occupation, higher socio-economic status of the family and social media effects [53]. On the other hand, the higher socioeconomic status families in developed countries with their greater awareness of the burden of obesity and its association with unhealthy diets, usually consume more vegetables and fruits than lower socio-economic class families, the members of which are also more likely to be sedentary [51].

The higher prevalence of O&O among private school pupils, pupils from higher socioeconomic class families and pupils with a television in their bedroom could be interrelated. Pupils from the more affluent families have greater access to energy surplus and calorie dense foods and drinks such as high calorie drinks, ice cream, and fast foods [51,54]. These families are also more able to afford the purchase of a television set for placement in the children’s rooms, just as they are more able to afford the high cost of private schools. In addition, private school pupils are more likely to be sedentary owing to greater involvement with indoor activities like videogames, *et cetera* [15].

The lack of a significant association between screen time and the prevalence of O&O in this study is unlike as in previous reports [15,44,55,56]. This could be due to under-reporting of viewing time by the parents as some of the children could have been watching the television without their parents' being aware of this. This could explain why the presence of televisions in the bedroom of pupils was a significant risk factor for O&O in bivariate analysis but screen time was not in this study [57]. Overall, however, the presence of electronic media in children's bedrooms is known to be associated with increased media use [57].

## Conclusion

We conclude that the prevalence of overweight and obesity among primary school children in semi-urban area was associated with gender, school type and family socioeconomic status. We therefore recommend the use of preventive programmes for the prevention of overweight and obesity among affluent children, especially female pupils attending private schools.

## Limitations of the study

Screen time could have been under or over estimated, being dependent on the recall ability of the parents. Screen time could also be underestimated because pupils might be watching the television or using other electronic devices in their bedrooms without the knowledge of their parents and this disparity could be more during non-school year. These factors could affect the reliability of the estimates of pupils’ screen time given by the parents.

## Supporting information

S1 Dataset The minimal dataset; “S1 Dataset” contains the following information: “school type”- private/public, “gender”- male/female, “Hours on screen”, “TV in bedroom”- Yes/No, “weight in kilogram”, “Height”- HT in cm, “BMI”–Body mass index, “BMI Z-score”, “SES”- Socioeconomic status score, “SES OYEDEJI”- socioeconomic status–classification by Oyedeji. (XLSX)
